# Aligned fibrous scaffolds promote directional migration of breast cancer cells *via* caveolin-1/YAP-mediated mechanosensing

**DOI:** 10.1016/j.mtbio.2024.101245

**Published:** 2024-09-14

**Authors:** Ping Li, Hanying Zhou, Ran Yan, Wei Yan, Lu Yang, Tingting Li, Xiang Qin, Yanyan Zhou, Li Li, Ji Bao, Junjie Li, Shun Li, Yiyao Liu

**Affiliations:** a Sichuan Provincial Key Laboratory for Human Disease Gene Study, Center for Medical Genetics, Sichuan Provincial People's Hospital, School of Life Science and Technology, University of Electronic Science and Technology of China, Chengdu, 610054, Sichuan, PR China; b Department of Pathology, Institute of Clinical Pathology, Key Laboratory of Transplant Engineering and Immunology, West China Hospital, Sichuan University, Chengdu, 610041, Sichuan, PR China; c Breast Surgery Department, Sichuan Clinical Research Center for Cancer, Sichuan Cancer Hospital & Institute, Sichuan Cancer Center, Affiliated Cancer Hospital of University of Electronic Science and Technology of China, Chengdu, 610054, Sichuan, PR China; d Traditional Chinese Medicine Regulating Metabolic Diseases Key Laboratory of Sichuan Province, Hospital of Chengdu University of Traditional Chinese Medicine, Chengdu, 610072, Sichuan, PR China; e Department of Urology, Deyang People's Hospital, Deyang, 618099, Sichuan, PR China

**Keywords:** Isotropic fibers, Matrix topology, Cell migration, Cav-1, Mechanotransduction

## Abstract

Tumorigenesis and metastasis are highly dependent on the interactions between the tumor and the surrounding microenvironment. In 3D matrix, the fibrous structure of the extracellular matrix (ECM) undergoes dynamic remodeling during tumor progression. In particular, during the late stage of tumor development, the fibers become more aggregated and oriented. However, it remains unclear how cancer cells respond to the organizational change of ECM fibers and exhibit distinct morphology and behavior. Here, we used electrospinning technology to fabricate biomimetic ECM with distinct fiber arrangements, which mimic the structural characteristics of normal or tumor tissues and found that aligned and oriented nanofibers induce cytoskeletal rearrangement to promote directed migration of cancer cells. Mechanistically, caveolin-1(Cav-1)-expressing cancer cells grown on aligned fibers exhibit increased integrin β1 internalization and actin polymerization, which promoted stress fiber formation, focal adhesion dynamics and YAP activity, thereby accelerating the directional cell migration. In general, the linear fibrous structure of the ECM provides convenient tracks on which tumor cells can invade and migrate. Moreover, histological data from both mice and patients with tumors indicates that tumor tissue exhibits a greater abundance of isotropic ECM fibers compared to normal tissue. And Cav-1 downregulation can suppress cancer cells muscle invasion through the inhibition of YAP-dependent mechanotransduction. Taken together, our findings revealed the Cav-1 is indispensable for the cellular response to topological change of ECM, and that the Cav-1/YAP axis is an attractive target for inhibiting cancer cell directional migration which induced by linearization of ECM fibers.

## Highlights

• Aligned fibrous scaffolds promote directional migration of breast cancer cells.
• Cav-1 is indispensable for the cellular response to the mechanical context of ECM fiber orientation.
• Downregulation of Cav-1 suppresses muscle invasion of cancer cells induced by isotropic ECM fibers.

## Graphical abstract

**Figure:**
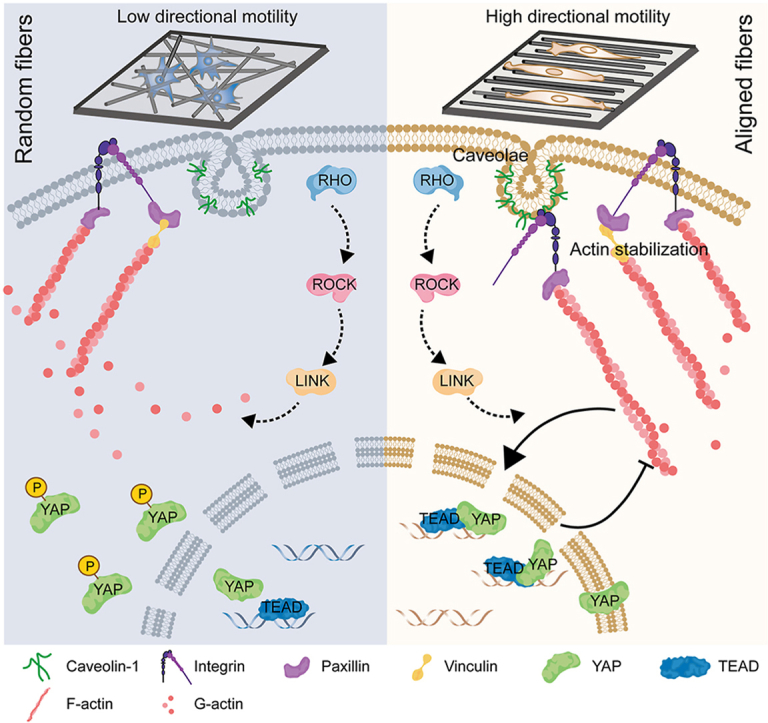

## 1 Introduction

Tumor resides in a highly complex, heterogeneous, and spatiotemporally dynamic tumor microenvironment (TEM), including different types of cells, biofactors, and ECM, which profoundly directs the malignant evolution of tumor [1,2].The ECM, a key component of the TEM, which not only provides physical support of surrounding cancer cells but also plays roles in mechanical and biochemical signal transduction. It is becoming increasingly evident that the physical properties of ECM, such as stiffness, viscoelasticity and topology, can affect the fate and function of tumor cells *via* mechanical signaling transductions. In particular, the topological cues of ECM (i.e., shape, arrangement, and dimensions) have considerable influence on cell adhesion, proliferation and differentiation. For instance, matrix viscoelasticity has been demonstrated to induce proliferation and invasion of hepatocellular carcinoma cells through an integrin-β1–tensin-1–YAP mediated mechanotransduction [3].

The structure of the ECM undergoes characteristic remodeling during tumor progression. For example, the ECM exhibits excessive deposition as a consequence of dysregulation in the production and degradation of ECM components, resulting in a considerable increase in ECM density and stiffness and a reduction in pore size. In addition, the ECM undergoes topological structural alterations, which are represented by the transformation from a fine and twisted anisotropic network into a highly aligned and isotropic and parallel architecture of collagen fibers due to fibroblast activation, heightened cell contraction, and upregulation of collagen cross-linking enzymes [4,5]. Previous studies remind us that the linearization of collagen may provide facilitation for directional migration of small and plastic tumor cells with stem like characteristics [6]. However, the mechanism by which tumor cells rapidly and efficiently respond to the topological remolding of ECM fiber network and orchestrate with biochemical signals to accelerate cell migration remain unclear.

Cells perceive biochemical and biomechanical signals from surrounding ECM *via* a variety of membrane proteins. For instance, cells sense the structural information of extracellular collagen through integrins, and regulate their motility through the outside-in mechanotransduction [7]. Caveolin-1 (Cav-1), a scaffolding protein located in cytomembrane, which is highly expressed in some kinds of tumors, and is highly associated with tumor associated progression including endocytosis, vesicular transport invasion and metastasis [8,9]. The previous study highlighted the significance of Cav-1 in the establishment of cell polarity during directional migration by coordinating Src kinase and Rho GTPase signaling [10]. Several studies have also shown that Cav-1 participates in mechanotransduction as a mechanical sensor in response to a range of mechanical stimuli, including membrane stretching, shear stress, and ECM stiffness [[11], [12], [13]]. For example, fluid shear stress enhances caveolin-dependent integrin β1 internalization and recycling resulting in promoting directional migration of cancer cells [14]. The involvement of Cav-1 in vesicular transport in endocytosis, exocytosis and cytoskeleton remodeling is the possibly reason for its activation of mechanotransduction. However, it remains unclear how Cav-1 is involved in the response to matrix structural remodeling of the ECM during tumor progression. Therefore, it is meaningful to investigate how Cav-1 responds to the topology cues of the ECM and benefits locally invade and metastasize.

Here, we used polycaprolactone electrospinning to fabricate nanofibrous substrates with either random or aligned networks. Subsequently, we conducted a comprehensive investigation into the cellular mechanisms underlying the sensing of changing topographical cues and their subsequent modulation of cell migration. In this study, we demonstrated that cancer cells could responded the topological cues to orchestrate cytoskeletal rearrangement and directed migration. The expression of Cav-1 in tumor cells cultured on aligned fibers was upregulated, resulting in integrin β1 internalization and F-actin polymerization. Consequently, this facilitated the translocation of YAP to the nucleus and cell migration. These findings suggested that Cav-1 plays an important role in the persistence migration of cancer cells, and the Cav-1/YAP signaling axis could be a potential target for cancer metastasis therapy.

## 2 Materials and methods

### 2.1 Cell culture

The triple negative human breast cancer cell line MDA-MB-231 was purchased from the Cell Bank of Type Culture Collection of Chinese Academy of Sciences (Shanghai, China). Cells were maintained at 37 °C in Leibovitz's L-15 (Thermo Fisher Scientific, USA) culture medium supplemented with 10 % fetal bovine serum (FBS) and 1 % penicillin-streptomycin. Cells were passaged every five days and were harvested using 0.25 % trypsin-EDTA (Thermo Fisher Scientific).

### 2.2 Antibodies and reagents

The following antibodies were used: anti-paxillin antibody (Cell Signaling Technology, USA), anti-caveolin-1 antibody (Cell Signaling Technology), anti-integrin antibody (BD Biosciences, USA). The TRITC/FITC-conjugated phalloidin was purchased from Sigma-Aldrich (USA). Cytochalasin D (Cyto D) (Sigma-Aldrich, USA) and jasplakinolide (Jasp) (Sigma-Aldrich) were dissolved at 10 mM in DMSO and stored at −20 °C until use. Inhibitor solutions were diluted in fresh medium and used to replace medium on cells.

### 2.3 Plasmids and transfection

pmCherry-paxillin were obtained from Addgene (USA). Lipofectamine® LTX (Invitrogen, USA) was used for transient transfections according to the manufacturer's protocol.

### 2.4 Electrospinning scaffold preparation

Electrospinning scaffolds were prepared by electrospinning as previously described [15]. Briefly, a 12 % (by weight) poly-ε-caprolactone (PCL) solution was prepared by dissolving in 34–35 °C dichloromethane with continuous stirring. After cooling to room temperature, the solution was placed in a 60-cc syringe with a 22-gauge blunt-tip needle and electrospun using a high-voltage DC power supply set to 18 kV and −3 kV, a tip-to-substrate distance of 15 cm and a flow rate of 0.8 mL/h. Rotating speed of random (RF) and aligned (AF) fibers were 600 rpm and 3000 rpm, respectively. Electrospinning was conducted in a chamber in which the relative humidity was maintained above 44 %. The electrospun fibers were deposited onto rollers for 2 h; the fiber sheet was then placed in a vacuum overnight to ensure the removal of residual solvent. 1 × 1 cm^2^ samples were cut and glued at the edges on glass coverslips for imaging analysis.

### 2.5 Scanning electron microscopy

Cells were cultured on random/aligned fibers for 24 h, then fixed with a solution of glutaraldehyde 3 % in 100 mM cacodylate buffer and dehydrated in a series of increasing concentrations of ethanol. Samples were mounted on aluminum stubs, sputter-coated with palladium and imaged using a Benchtop Scanning Electron Microscope (ZEISS, Germany).

### 2.6 Micropattern stamps for microprinting

A silicon master with the desired micropattern design was fabricated using standard lithographic techniques [16]. A PDMS stamp was prepared by mixing PDMS (SYLGARD 184, DOW Corning, Midland, IL, USA) and a cross-linking agent in a 10:1 ratio. The mixture was degassed in the desiccator to remove any trapped air bubbles and then cured at 85 °C for 2 h. The stamps were then peeled off from the silicon wafer and were cut into small pieces. The PDMS stamps were then cleaned by sonication (Branson) for 20 min and washed in 50 % ethanol solution before drying under nitrogen flow followed by plasma cleaning for 2 min (Harrick Plasma). The micropatterned areas of the PDMS stamps were soaked in fibronectin (FN) (BD, USA) solutions (40 μg/mL) in PBS and allowed to set for 1 h at room temperature, the FN solution was removed and the stamps were dried at 37 °C. The PDMS stamp was turned over so that the patterned surface was facing the nontreated hydrophobic dish (Jet, Guangzhou, China), and gently placed on the center of the dish and left for 30 min at room temperature. The stamp was carefully removed. Dishes were then treated with 1 % Pluronic F-127 (Sigma-Aldrich) for 1 h to block uncoated regions. Fluorescein isothiocyanate (Sigma-Aldrich) labeled FN was used to check the integrity of the micropatterns.

### 2.7 Western blot analysis

Cells were washed three times with ice-cold PBS solution and lysed in RIPA buffer (Beyotime Biotechnology, China) supplemented with Halt protease and phosphatase inhibitor cocktail (Thermo Fisher Scientific). Equal amounts of protein were separated by SDS-PAGE on 4 %–12 % Bis-Tris gels, and then blotted onto a PVDF membrane (Merck Millipore, USA). Membranes were probed with primary antibodies and blocked with 5 % milk/TBST for 1 h at room temperature before incubation with primary antibodies overnight at 4 °C. After washing with TBS containing 0.2 % Tween-20, the membranes were incubated with HRP-conjugated secondary antibodies for rabbit or mouse IgG. The membranes were then washed with TBS containing 0.2 % Tween-20 and incubated with chemiluminescent substrates. Primary antibodies were diluted at 1:1000. Secondary antibodies were diluted at 1:5000. Membranes were developed using the ECL Plus chemiluminescent substrate kit (Solarbio, China).

### 2.8 Immunofluorescence staining

Cells grown on electrospun fibers or patterned dishes were fixed with 4 % formaldehyde for 15 min at room temperature, permeabilized with 0.4 % Triton-X 100 (Biosharp, China) for 20 min, and blocked with 1 % bovine serum albumin (BSA) (Solarbio) for 1 h. Cells were then incubated overnight at 4 °C in 2.5 % BSA with primary antibodies, and stained with Alexa-conjugated secondary antibodies (1:400) for 2 h at room temperature. Images were captured using a laser confocal microscope (LSM800, ZEISS, Germany) or an Eclipse Ti2 microscope (Nikon, Japan) and analyzed using Fiji Image J software.

### 2.9 Image analysis

The cancer cell boundary was manually defined based on the image using the F-actin signals as the cell edge. The cell basal area, major axis length and minor axis length and were measured using the regionprops function in MATLAB. The aspect ratio of cancer cells was computed as: majoraxislength/minoraxislength.

### 2.10 Time-lapse microscopy

The plasmid pmCherry-paxillin was transiently transfected into cells to evaluate focal adhesion (FA) dynamics. A confocal laser scanning microscope (LSM800) was used for imaging. Images were captured continuously for 5 min at 1.5 s intervals to record cell behaviors. The motility of MDA-MB-231 cells was performed using an Eclipse Ti2 microscope (Nikon) with the Hoechst staining. The positions of all cells were recorded and used to draw the track. Image J software was used to calculate the Euclidean distance (d_Euclid_), average cell velocity, accumulated distance (D_accum_) and persistence of each group. The persistence of cell motility in a preferred direction was assessed by calculating a directionality for each cell, defined as $d_{Euclid}/D_{accum}$, persistence = 1 indicates that the cells moved along a straight line.

### 2.11 Animal studies

Five-week-old female NCG severe immunodeficiency mice were purchased from GemPharmatech (China), and were acclimated for one week. Ten mice were injected subcutaneously with 100 μL of Matrigel (Corning, USA) containing 1 × 10^6^ MDA-MB-231 cells or shCav-1 MDA-MB-231 cells. Each group consisted of 5 mice. The experiment was terminated at week 7 (three mice per group) and week 9 (two mice per group). Histochemical and immunohistochemical experiments were performed on tumor sections. The animal protocols complied with animal welfare laws and were authorized.

### Ethical statement

All procedures were performed following the protocol approved by the Institutional Animal Care Committee of the University of Electronic Science and Technology of China.

### 2.12 Statistical analysis

Each experiment was repeated at least three times. All the data are expressed as the mean ± standard deviation (SD) using GraphPad Prism software (version 8.0, USA). Statistical analyses between two groups were performed using the Student's *t*-test, and one-way ANOVA test with Tukey's post-test analysis was used for multigroup comparisons. Significant differences between groups were considered when *P* < 0.05 (∗), *P* < 0.01 (∗∗) or *P* < 0.001 (∗∗∗), while differences were considered remarkably statistically significant when *P* < 0.0001 (∗∗∗∗).

## 3 Results

### 3.1 Substrate topography modulates cancer cell morphology and migration

**Fig. 1:**
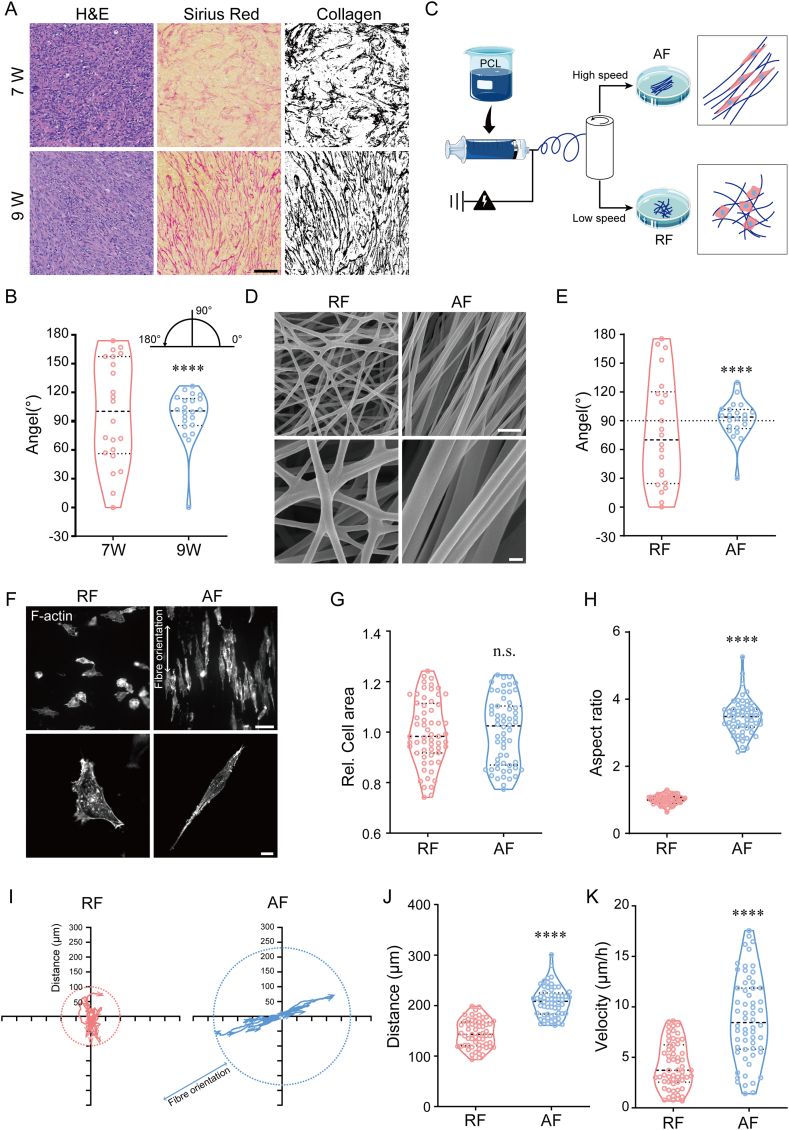
**The orientation of electrospun fibrous matrices dictate cell morphology.** (A) Representative images of tumor sections stained with SiriusRed and H&E. Scale bar is 50 μm. (B) Quantification of the collagen fiber orientations in tumor tissue. (C) Schematic illustration of the fabrication of aligned/random electrospun nanofibers. (D) Representative scanning electron microscopy images of random and aligned electrospun fibers (RF: random fibers; AF: aligned fibers). Scale bar is 2 μm in upper image and 200 nm in lower image. (E) Quantification of the electrospun fibers orientation. (F) Representative images of cell morphology. Cell were cultured on RF and AF substrates and stained with phalloidin. Scale bar is 20 μm in upper image and 10 μm in lower image. (G–H) Quantification of cell area and aspect ratio. (n ≥ 50). (I) Representative tracks of cells cultured on RF or AF substrate. n = 15, cells were tracked for 12 h. (J) Average motility distance was quantified from cell tracks. (n ≥ 50) (K) Average motility velocity was quantified from cell tracks. (n ≥ 50). The middle line shows medians, upper and lower lines as 25th and 75th percentiles, each datapoint is displayed as a dot, in (B, E, G, H, J, K). ∗∗∗∗*P* < 0.0001.

To investigate the changes in collagen fiber morphology during tumor development, we characterized the fiber orientation of tissue sections at early and late stages (7 and 9 weeks). Tissues were stained with hematoxylin and eosin (H&E) and Sirius red (Fig. 1A). In the early stage of tumor development, the reticulated collagen fibers are arranged in a disordered manner, whereas by the time the tumor has progressed to the mid to late stage, the fibers show a parallel and ordered arrangement, and this reorganization of collagen fibers provides a convenient trail for invasion and migration of the tumor cells (Fig. 1A and **B**). To investigate the potential role of isotropic fiber networks in cancer cell behaviors, we employed electrospinning technique to fabricate PCL biomimetic materials with different fiber orientations. Substrates with random (RF) and aligned (AF) fibers were used to mimic early or advanced tumor collagen fiber organizations (Fig. 1C). Scanning electron microscopy revealed that random and aligned electrospun PCL fibers were successfully fabricated (Fig. 1 D & **E**) with the orientations that similar to those of natural collagen fibers in early and late stages of tumors, respectively (Fig. 1. B). Subsequently, breast cancer MDA-MB-231 cells were seeded on RF and AF substrates and stained with FITC-phalloidin to observe the cell morphology generally (Fig. 1F). Cancer cells cultured on AF substrate extended a spindle-like shape with F-actin cytoskeletal bundles preferentially arranged along the fiber orientation, whereas cells cultured on RF showed a polygonal morphology. Spreading area and aspect ratio were used to evaluate the morphological features of single cells that cultured on two types of fiber networks. We found a dramatic increase in the aspect ratio of cells cultured on AF substrate than those cultured on RF (Fig. 1H), while cells cultured on both two types of fiber networks have similar cell area (Fig. 1G). These phenomena revealed that fibers with different arrangement features (parallel or random orientation) affect the morphological remodeling of cancer cells. Next, live-cell imaging was used to track the cell movement on aligned and random fibers, and to assess the cell motility. Cells cultured on AF substrate exhibited a fiber-oriented direction, longer migration distance and faster motility (Fig. 1I–K), suggesting that the ordered fibrous structure affected not only the migration direction but also the velocity.

### 3.2 Actin filament assembly is essential for directional cell migration on aligned fibrous substrates

**Fig. 2:**
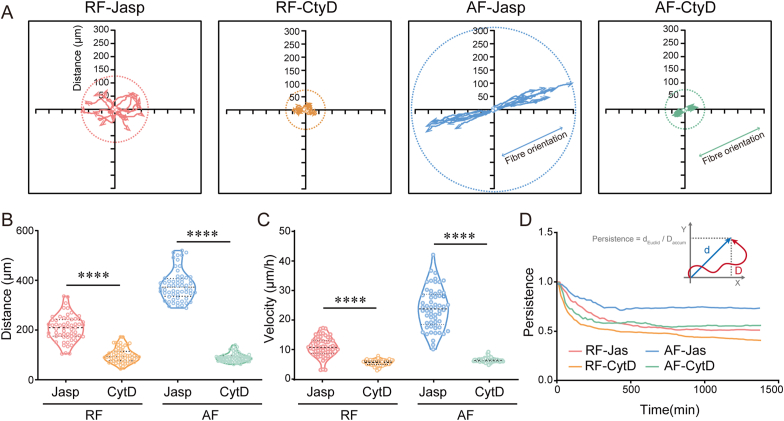
**The aligned fibers promote MDA-MB-231 cell migration through actin polymerization.** (A) Motility track plots of cells cultured on random fibers (RF) or aligned fibers (AF) substrates. which were treated with jasplakinolide (Jasp, 0.05 μM) and cytochalasin D (CtyD, 1 μM), respectively. (15 representative traces per condition were shown; cells were followed for 12 h). (B–C) Average motility distance and velocity were quantified (n ≥ 50). (D) Motility persistence was analyzed and plotted as a function of time. The middle line shows medians, upper and lower lines as 25th and 75th percentiles, each datapoint is displayed as a dot, in (B, C). ∗∗∗∗*P* < 0.0001.

The integrity and dynamics of the actin cytoskeleton plays a crucial role in cellular processes such as cell motility, endocytosis, cytokinesis, organelle transport, etc. [17]. In particular, actin filaments generate forces that drive cell shape changes and migration [18,19]. With the observation of significant cellular morphological remodeling of cells on RF and AF substrates (Fig. 1F), we next manipulated actin cytoskeleton dynamics to investigate the effect of actin filaments on cell migration through pharmacological approaches. MDA-MB-231 cells were treated with actin polymerizing reagents (jasplakinolide) or depolymerizing reagents (cytochalasin D) and time-lapse imaging was performed (Fig. 2A). Exposure to cytochalasin D resulted in a decrease in cancer cell migration, whereas moderate polymerization and stabilization of the actin filament by jasplakinolide promoted cell migration with increased distance, velocity and persistence (Fig. 2B–D). Furthermore, polymerization or disassembly of the actin filament did not affect the direction of cell motility on the substrates with random or aligned fibers networks (Fig. 2A). The results indicate that actin cytoskeleton rearrangement induced by substrate topography is necessary for the directional migration of cancer cells.

### 3.3 Aligned fibers stimulated Cav-1 expression and stress fiber formation to facilitate persistence migration

**Fig. 3:**
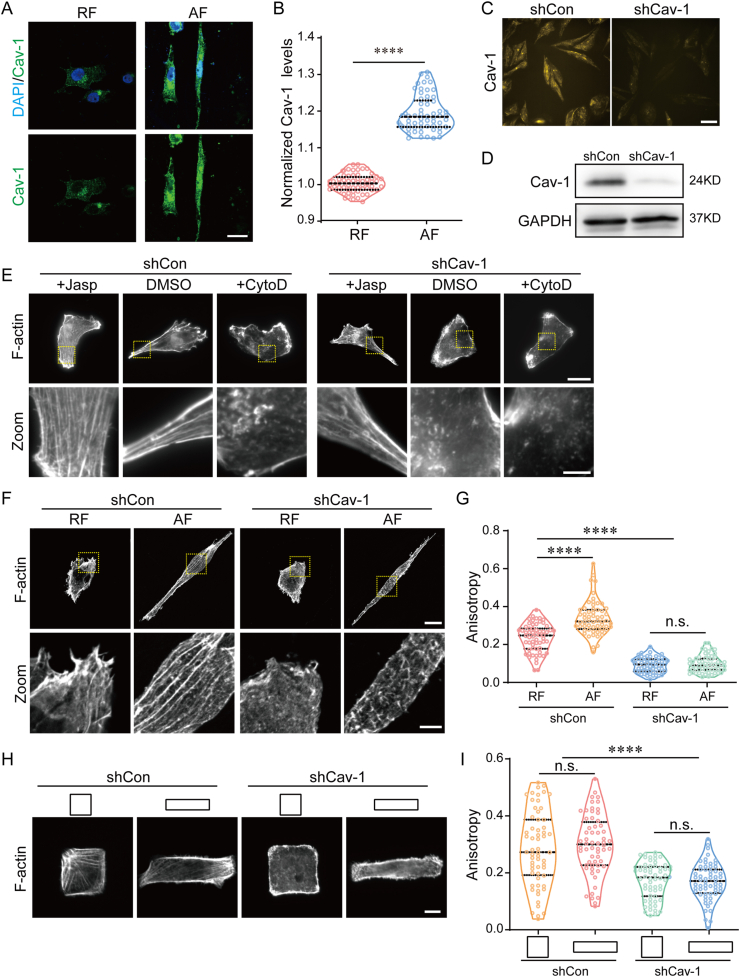
**Fiber orientation variation influences actin organization in a Cav-1 dependent manner.** (A) Representative images of cells plated on random fibers (RF) or aligned fibers (AF) substrates. Nuclear were stained with Cav-1 (green) and DAPI (blue). Scale bar is 20 μm. (B) Quantification of Cav-1 expression levels by immunofluorescence staining. (n ≥ 50). (C–D) Silence efficiency of Cav-1 were analyzed by immunofluorescence and western blot, respectively. Scale bar is 50 μm. (E) Representative images of stress fiber in shCon and shCav-1 cells grown for 24 h and treated with Jasp and CtyD, respectively. The bottom row shows zoomed views of the F-actin ROI. Scale bar is 20 μm in upper image and 5 μm in lower image. (F) Representative images of stress fiber in shCon or shCav-1 cells cultured on RF or AF substrates. F-actin were stained with phalloidin. Scale bar is 20 μm in upper image and 5 μm in lower image. (G) Quantification of stress fiber anisotropy of cells cultured on RF or AF substrates (n ≥ 50). (H) Representative images of stress fiber in shCon or shCav-1 cells cultured on micropatterns with different shapes. Scale bar is 10 μm (I) Quantification of stress fiber anisotropy of cells cultured on micropatterns with different shapes (n ≥ 50). The middle line shows medians, upper and lower lines as 25th and 75th percentiles, each datapoint is displayed as a dot, in (B, G, I). ∗∗∗∗*P* < 0.0001.

Cancer cells could regulate the level of Cav-1 to respond to biochemical or biomechanical stimuli. Sometimes the cellular level of Cav-1 is associated with the diversity of cell morphology. Considering that the arrangement of substrate fiber directly affects the morphological features of cancer cells, we wondered whether Cav-1 could respond to the changes of ECM topological cues. Immunostaining results showed that MDA-MB-231 cells cultured on AF substrate had a relatively higher level of Cav-1 (Fig. 3A and **B**). To investigate the role of Cav-1 in fiber arrangement stimulated actin network remodeling and cell migration ability, we used short hairpin RNA (shCav-1) to silence Cav-1 expression in MDA-MB-231 cells (Fig. 3C and **D**). Actin staining showed that knockdown of Cav-1 affected actin polymerization and exhibited disrupted F-actin filament bundles in jasplakinolide, cytochalasin D and no actin assemble/de-assemble treated groups (Fig. 3E), and that jasplakinolide treated shCav-1 cells still maintained a considerable amount of actin stress fibers (Fig. 3E). Since Cav-1-deficient cells displayed a damaged stress fiber, we wanted to determine the correlation between Cav-1 expression and actin polymerization induced by the AF substrate. Phalloidin staining showed that cells with silenced Cav-1 expression cultured on AF substrate showed impaired stress fibers formation with anisotropic and thin actin filaments (Fig. 3F and G), with a similar fibroblast-like cell morphology. To further verify the critical role of cell shape and Cav-1 in actin network construction, square micropatterns with different aspect ratios were used to mimic passive cellular morphological changes. Rectangular shaped cancer cells showed stress fibers through the long axis, while Cav-1 silenced cells exhibited significant impaired stress fibers formation (Fig. 3H and **I**). These results suggested that Cav-1 expression is indispensable for topological cue-induced actin cytoskeleton remodeling.

**Fig. 4:**
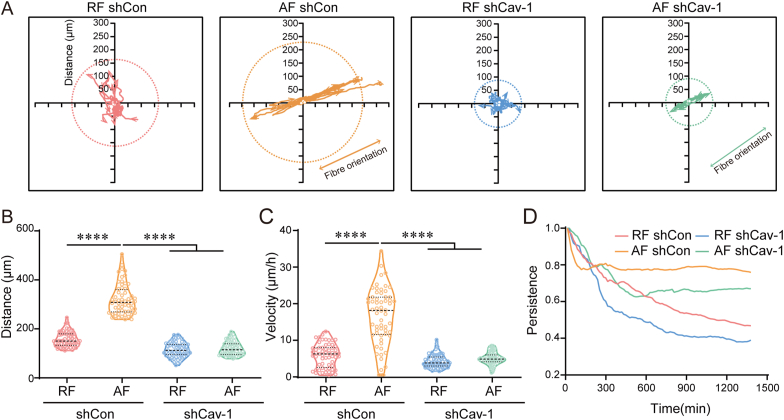
**Cav-1 is required for cells directional migration induced by fibers orientation.** (A) Motility track plots of shCon or shCav-1 cells cultured on random fibers (RF) or aligned fibers (AF) substrates, respectively. (15 representative traces per condition were shown; cells were followed for 12 h). (B–C) Average motility distance and velocity were quantified (n ≥ 50). (D) Motility persistence was analyzed and plotted as a function of time. The middle line shows medians, upper and lower lines as 25th and 75th percentiles, each datapoint is displayed as a dot, in (B, C). ∗∗∗∗*P* < 0.0001.

Since shCav-1 cells grown on aligned fibers showed an abnormal actin stress fiber network, we wondered whether this cytoskeleton defect induced by Cav-1 silence was involved in cancer cell migration and motility. Cell migration tracking experiment was performed, and the data showed that Cav-1 silence did not affect the direction of cell movement, which was determined by the orientation of the substrate fiber (Fig. 4A). While shCav-1 cells cultured on AF and RF fiber substrates exhibited shorter migration distance, slower velocity and weaker persistence (Fig. 4B–**D**). These observations suggest that Cav-1 is responsible for the enhanced cell motility and directional persistence which induced by aligned electrostatic filaments.

### 3.4 Cav-1 is required for integrin β1 internalization and FAs assembly

**Fig. 5:**
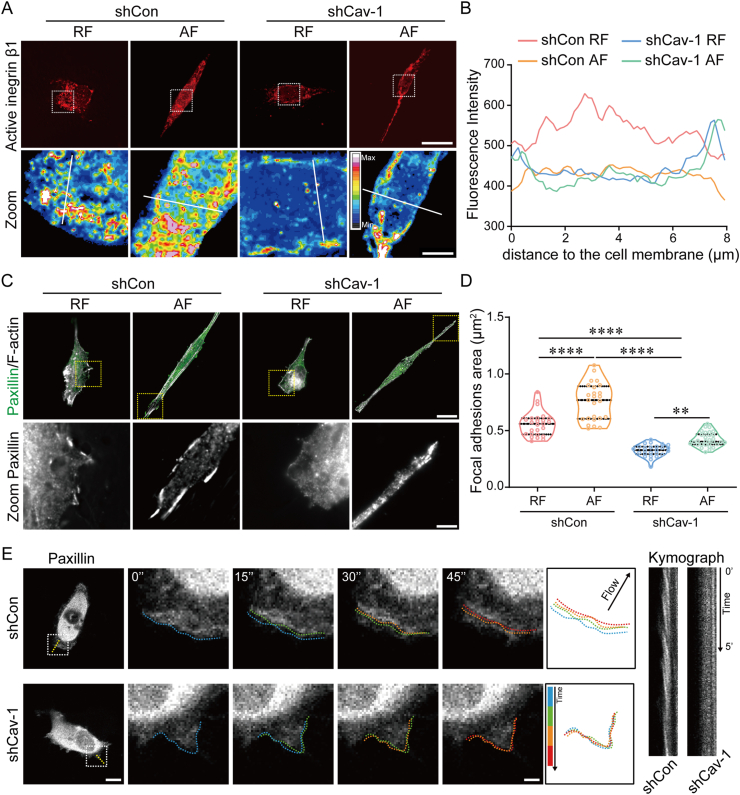
**Cav-1 is essential for the organization of nanofibers orientation-induced stress fiber formation and focal adhesion assembly.** (A) Representative immunofluorescence images of integrin β1 in cells cultured on random fibers (RF) or aligned fibers (AF) substrates. Scale bar is 20 μm in upper image and 5 μm in lower image. (B) Quantification of the fluorescence intensity across the white line in (A). Fluorescence intensity represent active integrin β1. (C) Representative immunofluorescence images of paxillin in cells cultured on RF or AF substrates. Stained with paxillin (green) and phalloidin (gray). Scale bar is 20 μm in upper image and 5 μm in lower image. (D) Quantification of FA number of cells (n ≥ 20). (E) Cells were transient transfected with pmCherry-paxillin. Time-lapse photography and kymograph was used to observe FA assembly in shCon or shCav-1 cells. Colored boundary outline marks the temporal changes of the edge of paxillin signaling. Scale bar is 10 μm in raw image and 2 μm in zoomed image. The middle line shows medians, upper and lower lines as 25th and 75th percentiles, each datapoint is displayed as a dot, in (D). ∗∗*P* < 0.01, ∗∗∗∗*P* < 0.0001.

Given our finding that shCav-1 cells experienced a dramatic shift in the cytoskeleton and an attenuated cell movement, we sought to further investigate how Cav-1 regulates directional cell migration. Prior to migration, following the formation of cellular extensions, such as filopodia and lamellipodia, in the direction of migration, the area of interaction with the ECM forms integrin-rich focal adhesion complexes that anchor the actin cytoskeleton to the matrix. Induced activation and traffic of integrins confers oncogenic properties to cancer cells, such as proliferation and migration, through altered adhesion dynamics and increased integrin signaling [20]. Considering that Cav-1 is essential for actin polymerization and persistent cell migration induced by AF substrate, the effect of Cav-1 on integrin β1 activation was further investigated. Immunofluorescence was used to visualize the localization and expression of integrin β1. Our results showed that activated integrin-β1 was distributed in the cytoplasm in shCon cells, whereas activated integrin β1 was mainly distributed on the surface of the cell membrane in shCav-1 cells. (Fig. 5A and **B**). Since the activation and endocytosis of integrin β1 serves as a trigger for focal adhesions (FAs) formation and assembly [21], paxillin staining was next used to estimate the role of Cav-1 in FAs formation. Control cells cultured on AF substrate extended oriented and large FAs along the actin filaments, whereas those cultured on RF formed smaller and fewer FAs. (Fig. 5C and **D**). To further observe the dynamics of FAs assembly, cells expressing mCherry-paxillin were traced with time-lapse imaging. Cells with Cav-1 expression showed a periodic and faster retrograde flow of paxillin, whereas silence of Cav-1 impaired the FAs assembly (Fig. 5E and Supplementary Figs. S1A–D). These results demonstrate that the Cav-1-dependent cytoskeleton is essential for integrin β1 internalization and FAs assembly.

### 3.5 Cav-1 dependent actin reorganization promotes YAP activity

**Fig. 6:**
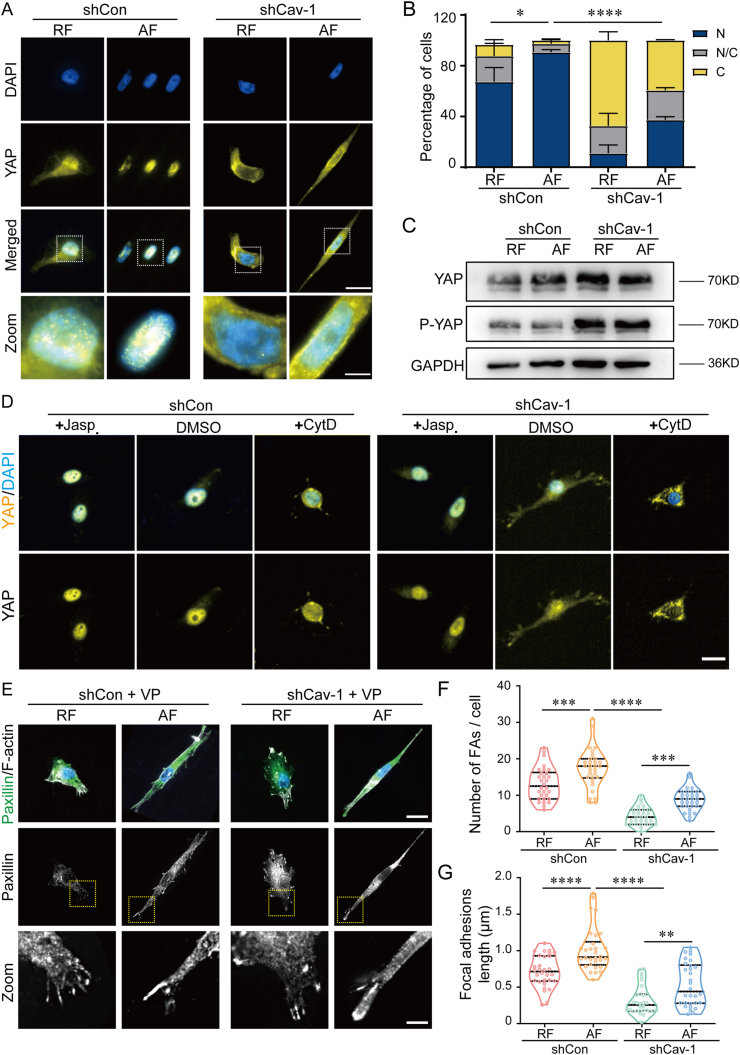
**Cav-1 dependent actin reorganization promotes YAP activity.** (A) Representative immunofluorescence images of YAP in cells cultured on random fibers (RF) or aligned fibers (AF) substrates. YAP (yellow) and DAPI (blue). Scale bar is 20 μm in raw image and 5 μm in zoomed image. (B) Percentage of cells from analysis YAP nuclear translocation. Predominantly nuclear YAP (N), predominantly cytosolic YAP (C), and nuclear-to-cytosolic distribution (N/C). (n ≥ 20). (C) Western blot of phosphorylated YAP and total YAP in shCon and shCav-1 cells cultured on RF or AF substrates. (D) Representative immunofluorescence images of YAP in cells with jasplakinolide, Cyto D or DMSO (control) treatment for 24 h. YAP (yellow) and DAPI (blue). Scale bar is 20 μm. (E) Representative immunofluorescence images of paxillin in cells treated with verteporfin (3 μM) for 24 h. Paxillin (green) and phalloidin (gray). Scale bar is 20 μm in raw image and 5 μm in zoomed image. (F) Quantification of FA number per cells. (n ≥ 30). (G) Quantification of FA length of cells. (n ≥ 30). Data are shown mean ± standard deviation (SD) in (B). ∗*P* < 0.05, ∗∗∗∗*P* < 0.0001. The middle line shows medians, upper and lower lines as 25th and 75th percentiles, each datapoint is displayed as a dot, in (F, G). ∗∗*P* < 0.01, ∗∗∗*P* < 0.001, ∗∗∗∗*P* < 0.0001.

YAP/TAZ, acting as mechanosensors, could respond to various mechanical cues to remodel cellular phenotypes and behaviors. The regulation of this YAP/TAZ-dependent mechanical process requires cytoskeleton integrity and involves various cytoskeletal and adhesive structures. To investigate whether the activity of YAP is affected by culture substrates with different fiber orientations, we analyzed the protein level and distribution of YAP. The cells cultured on the AF substrate showed a stronger YAP nuclear localization compared to the cells cultured on RF substrate. And Cav-1 knockdown blocked the enhanced nuclear localization of YAP in cells that cultured on AF (Fig. 6A and **B**). Meanwhile, silence of Cav-1 resulted in the upregulation of p-YAP (Fig. 6C). To further investigate whether this Cav-1-dependent YAP activity is associated with cytoskeleton remodeling, cells were treated with jasplakinolide (actin polymerization inducer) or cytochalasin D (actin polymerization inhibitor), respectively. In shCav-1 cells, it was observed that jasplakinolide treatment enhanced the nuclear localization of YAP, whereas cytochalasin D significantly inhibited the nuclear translocation of YAP (Fig. 6D). To gain further insight into the substrate fiber orientation induced and YAP mediated cell morphology and migration, we next observed and quantified FAs. Cells were then treated with verteporfin (YAP activity inhibitor). Interestingly, the length and number of FAs increased in cells cultured on AF substrate when YAP was inhibited, whereas Cav-1 silence inhibited the assembly of FAs in cells cultured on either RF or AF substrate (Fig. 6E–G). These results suggest that YAP is required for cancer cells to respond to substrate fiber orientation-induced changes in cell morphology and migration. In addition, micropatterns of squares and rectangles with an aspect ratio of 4:1 were used to mimic the cellular morphological changes induced by actin cytoskeleton rebuilding, and immunofluorescence staining conformed that Cav-1 and the cytoskeletal integrity were indispensable for YAP nuclear translocation (Supplementary Fig. S2).

### 3.6 Cav-1 promotes invasion of human breast cancer xenografts

**Fig. 7:**
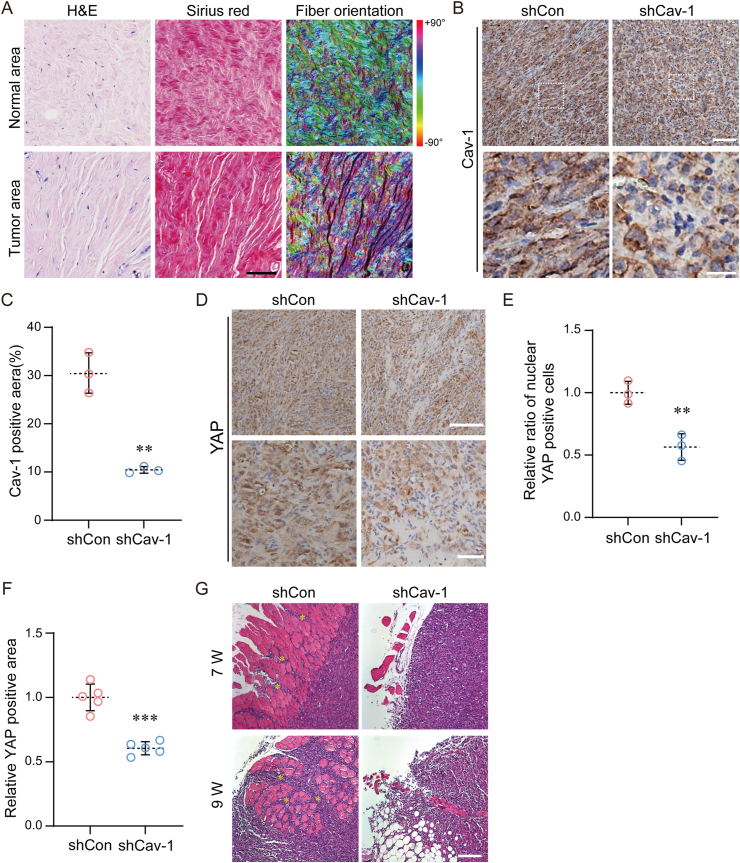
**Cav-1 and ECM fiber reorientation are essential for tumor invasion.** (A) Representative images of tumor sections from breast cancer patients stained with H&E and Sirius Red. Fibers orientations were showed with heat map. Scale bar is 200 μm. (B) Representative immunohistochemical analysis of Cav-1 expression in tissue sections. Scale bar is 200 μm in raw image and 50 μm in zoomed image. (C) Quantification of Cav-1 positive area in tissues. (n = 3) (D) Representative immunohistochemical analysis of YAP expression in tissue sections. Scale bar is 300 μm in raw image and 50 μm in zoomed image. (E) Quantification of the relative ratio of nuclear YAP-positive cells in tumor tissue (n = 3). (F) Quantification of the relative YAP-positive area of tumor tissue (n = 3). (G) Representative H&E staining images showing the muscle metastasis in the mouse model. Scale bar is 300 μm. Data are shown mean ± standard deviation (SD) in (C, E, F). ∗∗*P* < 0.01, ∗∗∗*P* < 0.001.

During tumor progression, invasion of tumor cells into surrounding tissues is a critical step. Our previous studies suggested that linearized fibers with an isotropic structure could promote tumor cell migration by providing tracks for tumor cells to move along. And this structural heterogeneity and orientation-driven promotion of tumor cell motility has also been demonstrated to be a Cav-1 indispensable process *in vitro*. More importantly, we observed similar organizational changes of collagen fibers between normal and tumor tissue from the same breast cancer patients. Collagen fibers in normal tissue showed a separated and vortex-like structure, whereas fibers in tumor exhibited continuous isotropic filaments (Fig. 7A). When Cav-1 was knocked down, the cytoplasm of cancer cells showed lower Cav-1 level and tumor tissues had a lower positive area of Cav-1 (Fig. 7B and **C**). Furthermore, tumor tissues with Cav-1 silence showed lower positive area and nuclear translocation of YAP (Fig. 7D–F). We then performed further studies to investigate the role of Cav-1 in tumor cell invasion *in vivo*. At 6 weeks, shCon tumor cells in mice had invaded the inner region of the surrounding muscle tissue, whereas shCav-1 tumor cells showed a much lower degree of invasion (Fig. 7E). After 8 weeks, shCon tumor cells had almost encapsulated the muscle tissue, whereas shCav-1 cells had not encapsulated the surrounding muscle or fat tissue (Fig. 7G). Taken together, the above evidence suggests that duo-therapeutic manipulation of the ECM fibers linearization and the presence of Cav-1 may provide a novel avenue to regulate cancer cell metastasis.

## 4 Discussion

**Fig. 8:**
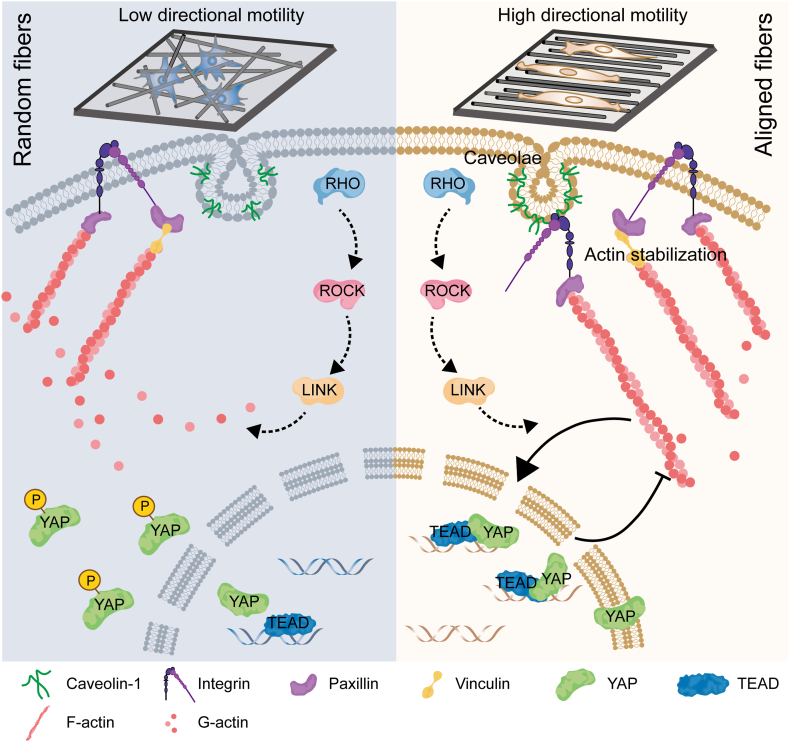
**Schematic illustration of the signaling pathway regulating cancer cell migration by electrospinning fiber orientation.** Cav-1 expression levels are regulated in response to random/aligned fiber orientation, which triggers downstream signaling. Specifically, Cav-1 induces actin polymerization, and the structural stability of stress fibers, which transduce extracellular signals to intracellular ones. YAP nuclear translocation is facilitated by parallel-arranged fibers, and the formation of cell focal adhesion is also promoted. Depending on the Cav-1/YAP mechanotransduction axis, isotropic ﬁbrous could eﬀectively induce changes in cell shape and accelerate directional migration.

Previous studies have shown that ECM assembly and remodeling occurred during tumor progression and metastasis [6,22]. The peritumoral collagen fibers gradually evolved from disordered to the orderly arrangement during breast tumor progression. Here electrospinning was used to mimic the topological evolution of fibrillar filaments during tumor progression. Our finding suggested that the aligned and isotropic fibers significantly affect the morphology and migration ability of cancer cells by remodeling actin cytoskeleton. Meanwhile, as a mechanically sensitive molecule, Cav-1 is indispensable in the response of fiber orientation and polymerization of actin stress fibers. In addition, aligned fibers and Cav-1 dependent stability of the actin cytoskeleton coordinately maintain the nuclear accumulation of YAP and promote FAs formation and cytoskeletal integrity to guide cancer cell migration (Fig. 8).

Physical cues (e.g., nano topography, matrix stiffness) at the cell-biomaterial interface have a strong influence on the cell phenotype and behavior [[23], [24], [25], [26], [27], [28], [29]]. Electrospinning has attracted considerable attention in the field of regenerative medicine due to its ability to create an environment that closely mimics the native ECM topological environment [[30], [31], [32]]. In this study, we used electrospinning technology to fabricate fibrous structures with different arrangements, and investigated the effect and mechanism of ECM arrangement on cell behavior and function. Our data demonstrated that the morphology of cells on scaﬀolds with different fiber arrangement varied signiﬁcantly, indicating that cell shape could reflect the change of topological cues. Next, we demonstrated that cancer cells could sense the extracellular topological cues to orchestrate cell migration. The ordered nanofibers provide convenience pathways and topological cues for cell migration, effectively guiding their movement and increasing the migration distance. Conversely, random nanofibers lack the ability to provide directional guidance for cell migration, resulting in limited directional displacements. These data suggest that the change in cellular morphology induced by ECM fibers arrangement is closely associated with cancer cell behavior and function. Note that in this study, cancer cells were cultured on the surface of 2D PCL fiber membranes with different arrangements. Therefore, the cells exhibited phenotypes and behaviors more similar to those cultured in 1D or 2D environments, although we observed that some cells have crawled into the interior of the multilayered fiber membrane. Here, we used 2D electrospun nanofiber membranes to mimic the evolutionary characteristics of fibrous arrangement in the tumor development, but there are still differences from the 3D microenvironment of the solid tumor. The application of hydrogels with adjustable orientation may be beneficial in further understanding the sensing and response of cancer cells to physical cues.

The expression and distribution of Cav-1, as well as Cav-1/caveolae mediated signaling, could be regulated by external mechanical cues. In vascular smooth muscle cells, cyclic mechanical stretch alters Cav-1 distribution, leading to a decrease in intracellular Cav-1 levels and an increase in its secretion into extracellular vesicles [12]. The ERK pathway is activated in a caveolae-dependent manner upon chronic shear stress exposure in endothelial cells [33]. In particular, Cav-1 is highlighted in mechanotransduction, pathophysiological processes and oncogenic cell transformation, tumorigenesis, and metastasis [[34], [35], [36], [37], [38]]. As an important mechanical signal-sensitive molecule, Cav-1 could respond to mechanical factors from the tumor microenvironment and regulate cell behavior through a series of molecular signal responses [39,40]. The previous study has shown that Cav-1 is involved in low shear stress-induced breast cell motility, FAs dynamics and adhesion of breast cancer cells [41]. Furthermore, substrate topography regulates differentiation of annulus fibrosus-derived stem cells *via* Cav-1 [42]. However, whether Cav-1 is involved in the response of cancer cell to topological structural changes of ECM, and by what mechanisms, remain unknown.

In this study, we showed that Cav-1 expression was increased in cancer cells that cultured on aligned fibers, suggesting that substrate topography might modulate cell behaviors and function in association with Cav-1. Further mechanistic studies showed that Cav-1 upregulation facilitates stress fiber formation, thereby increasing YAP nuclear location. YAP is generally considered to be a key mediator of biological effects controlled by cell shape, ECM elasticity and substrate topology [[43], [44], [45]]. Our data showed a clear correlation between YAP nuclear localization and Cav-1 expression in cells, and Cav-1 knockdown induced YAP cytosolic retention. We found that Cav-1 promoted YAP nuclear translocation in response to changes of fiber arrangement. We further confirmed the Cav-1-dependent positive regulation of YAP, invasion and metastasis *in vivo*. Taken together, these results demonstrated that Cav1-YAP plays a role in directional cell movement. Our study also supported the previous work that Cav-1 may act as an upstream positive regulator of YAP, determining the mechanical response to topological cues [11].

The actin cytoskeleton is a dynamic structure capable of adapting to mechanical changes in the environment by rearranging itself and is linked to the ECM through multiple sites of interaction, including integrins, FAs and cellular junctions. In addition to regulation of membrane tension, actin stress fibers directly associate with caveolae and play a critical role in the intracellular trafficking, endocytosis and exostosis of vesicles. Integrins and downstream focal adhesion complex proteins are recognized as mechanotransducers, responsible for sensing and converting mechanical signals into biochemical signals [[46], [47], [48], [49]]. The function of Cav-1 is highlighted in integrin-mediated ECM remodeling of tumor-associated fibroblasts [50], and in integrin-dependent invasion and metastasis of tumor cells [[51], [52], [53]]. These evidences have shown that caveolin, probably through interactions with integrins, can orchestrate mechanical signaling transduction events. However, the underlying mechanism by which Cav-1 and integrin regulate cell migration in the mechanical context of different nanofiber orientations is still unclear. In this work, we found that Cav-1 promotes stress fiber formation, thereby facilitating integrin β1 activation and endocytosis, which is associated with cell migration induced by aligned fibers.

## 5 Conclusions

In summary, we fabricated electrospun fibrous scaffolds with different orientations to mimic the evolution of fibrous structures in the stroma at different stages of tumor development and to investigate the role and mechanism of fiber arrangement in cell migration. When cancer cells were subjected to tensile forces that provided by parallel-arranged nanofibers, increased Cav-1 expression induced actin polymerization, promoted the nuclear translocation of YAP and FAs assemble, and enhanced directional migration. Our findings in this study suggest that the Cav-1/YAP axis may function as a key regulator of cell movement in the mechanical context of ECM fiber orientation, and provide a novel target for the prevention and treatment of cancer cell migration induced by collagen linearization in tumor tissue.

## Conflict of interest disclosure statement

The authors claim no conflicts of interest.

## CRediT authorship contribution statement

**Ping Li:** Writing – original draft, Validation, Investigation, Formal analysis, Data curation. **Hanying Zhou:** Validation, Investigation, Formal analysis, Data curation. **Ran Yan:** Investigation, Data curation. **Wei Yan:** Investigation, Data curation. **Lu Yang:** Visualization. **Tingting Li:** Methodology, Funding acquisition, Conceptualization. **Xiang Qin:** Methodology, Funding acquisition, Conceptualization. **Yanyan Zhou:** Methodology, Investigation. **Li Li:** Methodology, Investigation. **Ji Bao:** Writing – review & editing, Conceptualization. **Junjie Li:** Writing – review & editing, Resources. **Shun Li:** Writing – review & editing, Supervision, Funding acquisition, Conceptualization. **Yiyao Liu:** Writing – review & editing, Supervision, Funding acquisition, Conceptualization.

## Declaration of competing interest

The authors declare that they have no known competing financial interests or personal relationships that could have appeared to influence the work reported in this paper.

## Data Availability

Data will be made available on request.
