# Supplementary material for: Aligned fibrous scaffolds promote directional migration of breast cancer cells via caveolin-1/YAP-mediated mechanosensing
Source: Mater Today Bio. 2024 Sep 14;28:101245. doi: 10.1016/j.mtbio.2024.101245 (PMC11421348; doi:10.1016/j.mtbio.2024.101245)
Supplement: Multimedia component 1 [file mmc1.docx]

**Aligned fibrous scaffolds** **promote directional migration of breast cancer cells *via* caveolin-1/YAP-mediated mechanosensing**

Ping Li ^1,§^, Hanying Zhou ^1,§^, Ran Yan ^1^, Wei Yan ^1^, Lu Yang ^1^, Tingting Li ^1^, Xiang Qin^1^, Ji Bao ^2, *^, Junjie Li ^3, *^, Shun Li ^1 *^, Yiyao Liu ^1,4,5 *^

*^1^ Sichuan Provincial Key Laboratory for Human Disease Gene Study, Center for Medical Genetics, Sichuan Provincial People's Hospital, School of Life Science and Technology, University of Electronic Science and Technology of China, Chengdu, 610054, Sichuan, P. R. China.*

*^2^ Department of Pathology, Institute of Clinical Pathology, Key Laboratory of Transplant Engineering and Immunology, West China Hospital, Sichuan University, Chengdu 610041, Sichuan, P.R. China*

*^3^ Breast Surgery Department, Sichuan Clinical Research Center for Cancer, Sichuan Cancer Hospital & Institute, Sichuan Cancer Center, Affiliated Cancer Hospital of University of Electronic Science and Technology of China, Chengdu, 610054, Sichuan, P. R. China.*

*^4^ Traditional Chinese Medicine Regulating Metabolic Diseases Key Laboratory of Sichuan Province, Hospital of Chengdu University of Traditional Chinese Medicine, Chengdu 610072, Sichuan, P. R. China.*

*^5^ Department of Urology, Deyang People's Hospital, Deyang 618099, Sichuan, China.*

^§^These authors contributed equally to this work

***Corresponding authors**:

E-mail address: [baoji@scu.edu.cn](mailto:baoji@scu.edu.cn) (J. Bao), [lijunjie@scszlyy.org.cn](mailto:lijunjie@scszlyy.org.cn) (J. Li) [shunli@uestc.edu.cn](mailto:shunli@uestc.edu.cn) (S. Li), [liuyiyao@uestc.edu.cn](mailto:liuyiyao@uestc.edu.cn) (Y. Liu)

**Supplementary Figures**


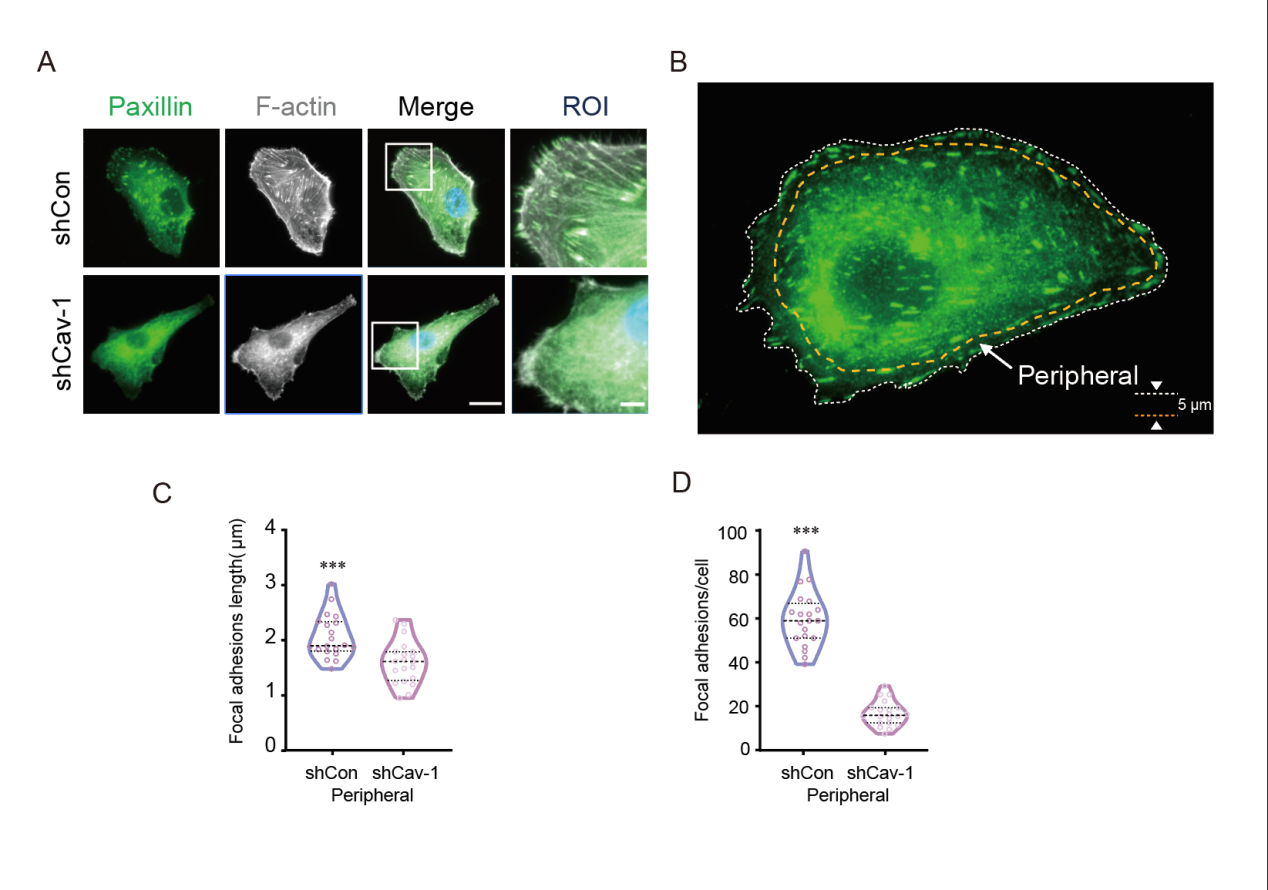


**Figure S1. Cav-1 is essential for focal adhesion assembly.** (A) Representative immunofluorescence images of paxillin distribution in shCon/shCav-1 MDA-MB-231 cells. Stained with paxilin (green) and phalloidin (gray). The right column shows zoomed views of the paxillin ROI (boxed in white in the paxillin images). Scale bar is 20 μm in raw image and 5 μm in zoomed image. (B) Spatial differences in FA morphology were detected by subdividing cells into peripheral and central (5 µm from the edge of the cell) regions. (C) Quantification of FA number (paxillin) for shCon/shCav-1 cells on RF and AF substrates (n ≥ 20). (D) Quantification of FA length (paxillin) for shCon/shCav-1 cells on RF and AF substrates (n ≥ 20).


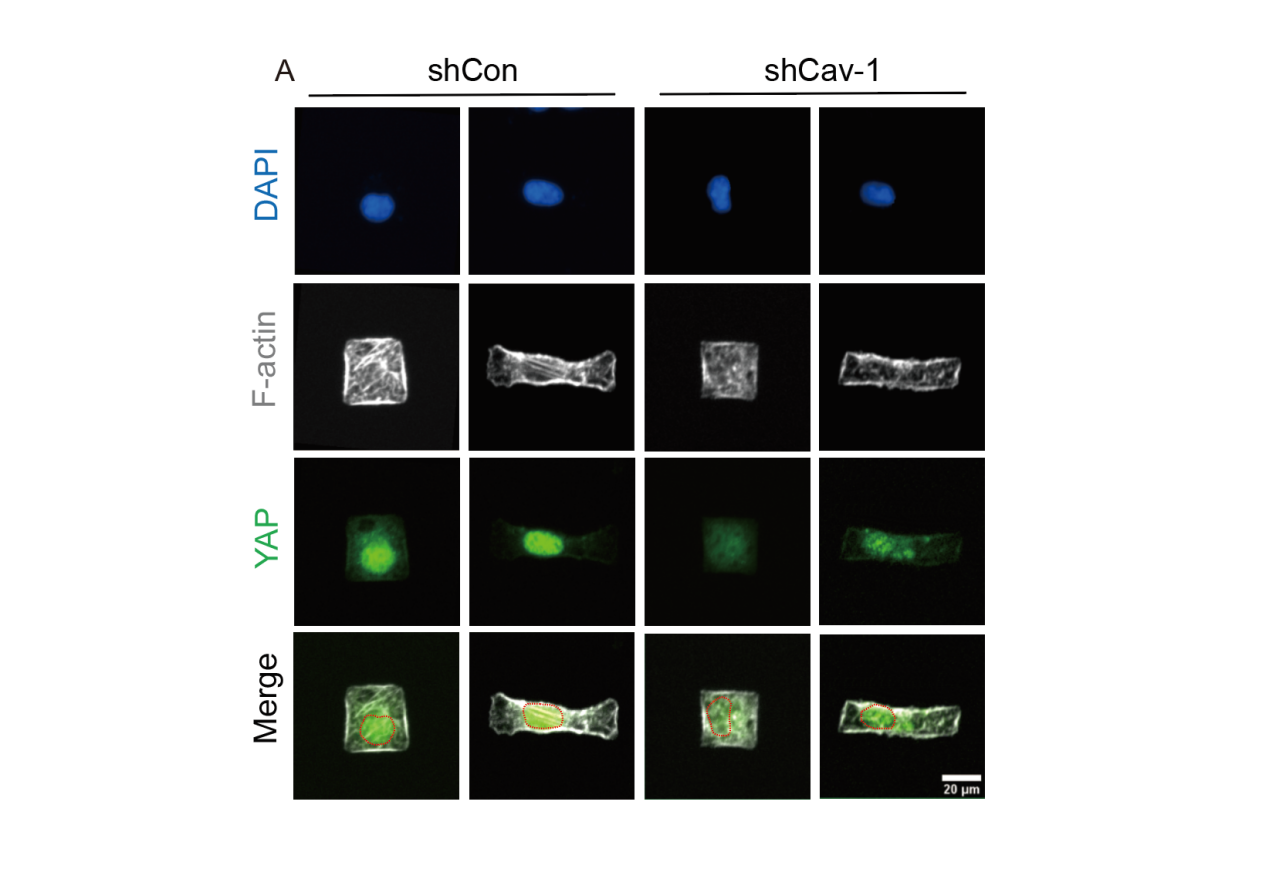


**Figure S2. Cav-1 is critical for YAP expression and nuclear localization.** (A) Representative immunofluorescence images of YAP distribution in shCon/shCav-1 MDA-MB-231 cells cultured on micropatterns with different shapes. Stained with YAP (green), DAPI (blue) and phalloidin (gray). The scale bar = 20 μm.
